# *cis*-{2,6-Bis[(di-*tert*-butyl­phosphan­yl)meth­yl]cyclo­hexyl-κ^3^ *P*,*C* ^1^,*P*′}chloridopalladium(II)

**DOI:** 10.1107/S1600536812047022

**Published:** 2012-11-24

**Authors:** Daniel Olsson, J. Marthinus Janse van Rensburg, Ola F. Wendt

**Affiliations:** a Centre for Analysis and Synthesis, Department of Chemistry, Lund University, PO Box 124, S-221 00 Lund, Sweden

## Abstract

The Pd^II^ atom in the title compound, [Pd(C_24_H_49_P_2_)Cl], has a distorted square-planar CClP_2_ coordination geometry with the *P*,*C*,*P*′-tridentate ligand forming two five-membered metallacycles. The cyclo­hexane ring is aligned with the Pd^II^ coordination plane due to C—H activation in an equatorial position, giving a tri-equatorial conformation of the cyclo­hexyl ring.

## Related literature

C(*sp* ^3^)—H activated (PCP)-complexes with catalytic performance in C—C coupling reactions were reported by Ohff *et al.* (1997 ▶); Sjövall *et al.* (2002 ▶); Nilsson & Wendt (2005 ▶); Olsson & Wendt (2009 ▶). Metal complexes with (PCP)-type ligands containing an aliphatic backbone have been reported for Rh (Kuznetsov *et al.*, 2006 ▶), Ni (Castonguay *et al.*, 2006 ▶; Pandarus & Zargarian, 2007 ▶), Pt (Olsson *et al.* 2007*a* ▶), Ir (Arunachalampillai *et al.*, 2009 ▶; Jonasson *et al.* 2011 ▶). The crystal structures of the bromide and iodide analogues of the title compound were determined by Sjövall *et al.* (2002 ▶) and Olsson *et al.* (2007*b* ▶).
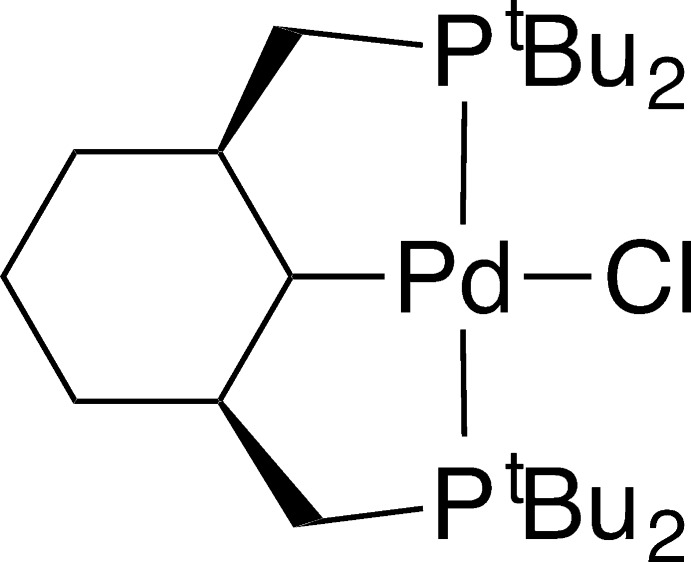


## Experimental

### 

#### Crystal data

[Pd(C_24_H_49_P_2_)Cl]
*M* *_r_* = 541.42
Monoclinic, 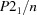

*a* = 11.9467 (2) Å
*b* = 14.6159 (2) Å
*c* = 15.5190 (3) Å
β = 100.339 (2)°
*V* = 2665.80 (8) Å^3^
*Z* = 4
Mo *K*α radiation
μ = 0.93 mm^−1^
*T* = 293 K
0.15 × 0.10 × 0.05 mm

#### Data collection

Oxford Diffraction XCalibur 3 diffractometer
Absorption correction: multi-scan (*CrysAlis RED*; Oxford Diffraction, 2006 ▶) *T*_min_ = 0.941, *T*_max_ = 1.000
26794 measured reflections
9297 independent reflections
6699 reflections with *I* > 2σ(*I*)
*R*_int_ = 0.024

#### Refinement

*R*[*F* ^2^ > 2σ(*F* ^2^)] = 0.028
*wR*(*F* ^2^) = 0.070
*S* = 0.96
9297 reflections
253 parameters
H-atom parameters constrained
Δρ_max_ = 1.28 e Å^−3^
Δρ_min_ = −0.56 e Å^−3^

### 

Data collection: *CrysAlis CCD* (Oxford Diffraction, 2006 ▶); cell refinement: *CrysAlis RED* (Oxford Diffraction, 2006 ▶); data reduction: *CrysAlis RED*; program(s) used to solve structure: *SHELXS97* (Sheldrick, 2008 ▶); program(s) used to refine structure: *SHELXL97* (Sheldrick, 2008 ▶); molecular graphics: *CrystalMaker* (*CrystalMaker*, 2011 ▶); software used to prepare material for publication: *SHELXL97*.

## Supplementary Material

Crystal structure: contains datablock(s) I, global. DOI: 10.1107/S1600536812047022/wm2700sup1.cif

Structure factors: contains datablock(s) I. DOI: 10.1107/S1600536812047022/wm2700Isup2.hkl

Additional supplementary materials:  crystallographic information; 3D view; checkCIF report

## Figures and Tables

**Fig. 1.**
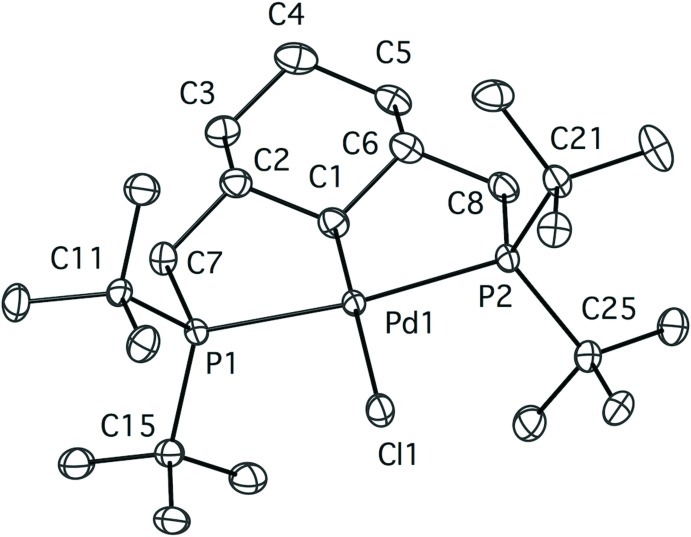
The molecular structure of (I) with atom labels (methyl groups labels omitted) and 40% probability displacement ellipsoids. H-atoms were omitted for clarity.

## supplementary crystallographic information

### Comment

In this study we report the crystal structure of {cis-1,3-bis[(di-*tert*-butylphosphanyl)methyl]cyclohexane}palladium(II) chloride, [PdCl(C_24_H_49_P_2_)], (I).

Compound (I) belongs to a family of C(*sp*^3^)—H activated (PCP)-complexes, showing interesting catalytic performance in C—C coupling reactions (Ohff *et al.*, 1997; Sjövall *et al.*, 2002; Nilsson & Wendt, 2005; Olsson & Wendt, 2009). Structural data for the corresponding bromide and iodide analogues have been reported previously (Sjövall *et al.*, 2002; Olsson *et al.*, 2007*b*).

Aromatic backbones are by far the most commonly occurring for palladium (PCP)-complexes, but complexes based on an aliphatic backbone are receiving increasing attention. Aliphatic (PCP)-type ligands that are coordinated to transition metals have been published recently for metals such as rhodium (Kuznetsov *et al.*, 2006), nickel (Castonguay *et al.*, 2006; Pandarus & Zargarian, 2007), platinum (Olsson *et al.* 2007*a*) and iridium (Arunachalampillai *et al.*, 2009; Jonasson *et al.* 2011).

In the structure of (I) the Pd^II^ atom exhibits a *pseudo*-square-planar coordination geometry (Fig. 1). Comparison to the analogous iodido and bromido complexes indicates the expected Pd—halogen bond lengths decrease. The Pd—P bond lengths are around 2.3 Å in all complexes with a *trans* orientation of the P atoms; in (I) the P1—Pd1—P2 angle is 166.495 (15) ° . The (PCP)-tridentate ligand and the Pd^II^ atom form two five-membered metalla rings. As is usually observed in these systems, the bis-chelating system displays two acute P—Pd—C1 angles of around 83–84°. Bond lengths are Pd1—Cl1, 2.4405 (4) Å, Pd1—P1, 2.3233 (4) Å, Pd1—P2, 2.3226 (4) Å and Pd1—C1, 2.0808 (16) Å.

The cyclohexane ring is aligned with the palladium coordination plane forming the usual tri-equatorial conformation (Fig. 1).

### Experimental

All procedures were performed under vacuum or nitrogen. The (PCP)H ligand was prepared according to the published procedure (Sjövall *et al.*, 2002). A solution of the ligand (0.536 g, 1.337 mmol) in 20 ml THF was mixed with a solution of PdCl_2_(PhCN)_2_ (0.500 g, 1.304 mmol) in 30 ml THF in a high-pressure glass vessel and the mixture was heated at 353 K for 8 h. Evaporation of all volatiles gave a crude, light yellow product in almost quantitative yield. Recrystallization from hexane gave 0.483 g (69%) of crystals suitable for X-ray crystallographic analysis. ^1^H-NMR (benzene-d_6_): δ 2.15–0.80 (m region, 13H, CH & CH_2_), 1.37 (m, 36H, coalesced virtual triplets). ^31^P{^1^H} NMR (benzene-d_6_): δ 70.6 (*s*).

### Refinement

The H atoms were positioned geometrically and treated as riding on their parent atoms with C–H distances of 0.93–0.97 Å and *U*_iso_(H) = 1.2*U*_eq_ - 1.5*U*_eq_. The highest difference peak in the Fourier map is located 1.25 Å from H26A and the lowest is located 0.60 Å from P2.

### Crystal data

**Table:**

[Pd(C_24_H_49_P_2_)Cl]	*F*(000) = 1144
*M**_r_* = 541.42	*D*_x_ = 1.349 Mg m^−^^3^
Monoclinic, *P*2_1_/*n*	Mo *K*α radiation, λ = 0.71073 Å
Hall symbol: -P 2yn	Cell parameters from 14595 reflections
*a* = 11.9467 (2) Å	θ = 2.2–33.0°
*b* = 14.6159 (2) Å	µ = 0.93 mm^−^^1^
*c* = 15.5190 (3) Å	*T* = 293 K
β = 100.339 (2)°	Prism, colourless
*V* = 2665.80 (8) Å^3^	0.15 × 0.10 × 0.05 mm
*Z* = 4	

### Data collection

**Table:**

Oxford Diffraction XCalibur 3 diffractometer	9297 independent reflections
Radiation source: Enhance (Mo) X-ray Source	6699 reflections with *I* > 2σ(*I*)
Graphite monochromator	*R*_int_ = 0.024
Detector resolution: 16.1829 pixels mm^-1^	θ_max_ = 33.0°, θ_min_ = 2.2°
ω scans	*h* = −18→18
Absorption correction: multi-scan (*CrysAlis RED*; Oxford Diffraction, 2006)	*k* = −20→22
*T*_min_ = 0.941, *T*_max_ = 1.000	*l* = −17→23
26794 measured reflections	

### Refinement

**Table:**

Refinement on *F*^2^	Primary atom site location: structure-invariant direct methods
Least-squares matrix: full	Secondary atom site location: difference Fourier map
*R*[*F*^2^ > 2σ(*F*^2^)] = 0.028	Hydrogen site location: inferred from neighbouring sites
*wR*(*F*^2^) = 0.070	H-atom parameters constrained
*S* = 0.96	*w* = 1/[σ^2^(*F*_o_^2^) + (0.040*P*)^2^] where *P* = (*F*_o_^2^ + 2*F*_c_^2^)/3
9297 reflections	(Δ/σ)_max_ = 0.004
253 parameters	Δρ_max_ = 1.28 e Å^−^^3^
0 restraints	Δρ_min_ = −0.56 e Å^−^^3^

### Special details

**Table:**

Geometry. All e.s.d.'s (except the e.s.d. in the dihedral angle between two l.s. planes) are estimated using the full covariance matrix. The cell e.s.d.'s are taken into account individually in the estimation of e.s.d.'s in distances, angles and torsion angles; correlations between e.s.d.'s in cell parameters are only used when they are defined by crystal symmetry. An approximate (isotropic) treatment of cell e.s.d.'s is used for estimating e.s.d.'s involving l.s. planes.
Refinement. Refinement of *F*^2^ against ALL reflections. The weighted *R*-factor *wR* and goodness of fit *S* are based on *F*^2^, conventional *R*-factors *R* are based on *F*, with *F* set to zero for negative *F*^2^. The threshold expression of *F*^2^ > σ(*F*^2^) is used only for calculating *R*-factors(gt) *etc*. and is not relevant to the choice of reflections for refinement. *R*-factors based on *F*^2^ are statistically about twice as large as those based on *F*, and *R*- factors based on ALL data will be even larger.

### Fractional atomic coordinates and isotropic or equivalent isotropic displacement parameters (Å^2^)

**Table:**

	*x*	*y*	*z*	*U*_iso_*/*U*_eq_	
Pd1	0.395006 (10)	0.557045 (8)	0.252150 (7)	0.01343 (4)	
Cl1	0.36623 (3)	0.67441 (3)	0.35789 (3)	0.02067 (8)	
P1	0.20462 (4)	0.53783 (3)	0.18983 (3)	0.01455 (8)	
P2	0.59247 (3)	0.55763 (3)	0.28541 (3)	0.01541 (8)	
C1	0.41983 (14)	0.46752 (12)	0.15303 (11)	0.0188 (3)	
H1	0.4226	0.5065	0.1021	0.023*	
C2	0.32136 (14)	0.40129 (11)	0.12327 (11)	0.0197 (3)	
H2	0.3176	0.3597	0.1722	0.024*	
C3	0.33987 (15)	0.34263 (12)	0.04548 (10)	0.0210 (3)	
H3A	0.2794	0.2977	0.0332	0.025*	
H3B	0.3356	0.3813	−0.0058	0.025*	
C4	0.45339 (16)	0.29375 (12)	0.06192 (11)	0.0268 (4)	
H4A	0.4642	0.2621	0.0091	0.032*	
H4B	0.4539	0.2485	0.1077	0.032*	
C5	0.55085 (15)	0.36146 (12)	0.08926 (10)	0.0215 (3)	
H5A	0.5551	0.4029	0.0411	0.026*	
H5B	0.6222	0.3283	0.1025	0.026*	
C6	0.53358 (15)	0.41664 (12)	0.16956 (11)	0.0211 (3)	
H6	0.5313	0.3731	0.2172	0.025*	
C7	0.20875 (14)	0.45253 (11)	0.10369 (11)	0.0196 (3)	
H7A	0.1461	0.4099	0.1016	0.024*	
H7B	0.2011	0.4826	0.0472	0.024*	
C8	0.63117 (14)	0.48257 (12)	0.20024 (11)	0.0204 (3)	
H8A	0.6460	0.5190	0.1513	0.024*	
H8B	0.6996	0.4486	0.2236	0.024*	
C11	0.11083 (13)	0.48541 (11)	0.26177 (10)	0.0172 (3)	
C12	0.16985 (16)	0.39565 (12)	0.29573 (12)	0.0255 (4)	
H12A	0.2458	0.4085	0.3254	0.038*	
H12B	0.1726	0.3554	0.2473	0.038*	
H12C	0.1280	0.3670	0.3357	0.038*	
C13	−0.00951 (14)	0.46389 (13)	0.21394 (12)	0.0252 (4)	
H13A	−0.0470	0.5197	0.1926	0.038*	
H13B	−0.0514	0.4347	0.2536	0.038*	
H13C	−0.0057	0.4238	0.1656	0.038*	
C14	0.10502 (15)	0.54682 (12)	0.34093 (11)	0.0242 (4)	
H14A	0.1807	0.5603	0.3709	0.036*	
H14B	0.0640	0.5158	0.3801	0.036*	
H14C	0.0666	0.6028	0.3214	0.036*	
C15	0.13876 (14)	0.64162 (11)	0.12899 (10)	0.0195 (3)	
C16	0.03613 (16)	0.62126 (13)	0.05651 (12)	0.0290 (4)	
H16A	0.0577	0.5776	0.0162	0.044*	
H16B	0.0114	0.6768	0.0258	0.044*	
H16C	−0.0248	0.5967	0.0822	0.044*	
C17	0.10405 (16)	0.71136 (12)	0.19252 (12)	0.0261 (4)	
H17A	0.1678	0.7241	0.2380	0.039*	
H17B	0.0428	0.6870	0.2179	0.039*	
H17C	0.0797	0.7668	0.1616	0.039*	
C18	0.23380 (16)	0.68321 (12)	0.08639 (12)	0.0271 (4)	
H18A	0.2561	0.6400	0.0461	0.041*	
H18B	0.2981	0.6975	0.1310	0.041*	
H18C	0.2065	0.7380	0.0556	0.041*	
C21	0.66048 (14)	0.50577 (12)	0.39318 (11)	0.0200 (3)	
C22	0.61384 (17)	0.40707 (12)	0.39274 (12)	0.0286 (4)	
H22A	0.6367	0.3732	0.3458	0.043*	
H22B	0.5323	0.4087	0.3846	0.043*	
H22C	0.6436	0.3780	0.4475	0.043*	
C23	0.79068 (15)	0.50157 (16)	0.40666 (12)	0.0321 (4)	
H23A	0.8133	0.4693	0.3587	0.048*	
H23B	0.8194	0.4703	0.4605	0.048*	
H23C	0.8209	0.5626	0.4092	0.048*	
C24	0.62365 (15)	0.55715 (12)	0.46951 (11)	0.0235 (3)	
H24A	0.5421	0.5601	0.4604	0.035*	
H24B	0.6543	0.6180	0.4726	0.035*	
H24C	0.6515	0.5255	0.5233	0.035*	
C25	0.65819 (14)	0.67117 (11)	0.26587 (11)	0.0199 (3)	
C26	0.77759 (15)	0.66373 (13)	0.24310 (13)	0.0304 (4)	
H26A	0.7752	0.6242	0.1934	0.046*	
H26B	0.8290	0.6390	0.2922	0.046*	
H26C	0.8032	0.7234	0.2294	0.046*	
C27	0.66061 (16)	0.73466 (12)	0.34422 (12)	0.0268 (4)	
H27A	0.5856	0.7391	0.3580	0.040*	
H27B	0.6859	0.7943	0.3302	0.040*	
H27C	0.7119	0.7105	0.3938	0.040*	
C28	0.57787 (16)	0.71338 (13)	0.18684 (12)	0.0276 (4)	
H28A	0.5027	0.7187	0.2001	0.041*	
H28B	0.5758	0.6748	0.1365	0.041*	
H28C	0.6052	0.7729	0.1747	0.041*	

### Atomic displacement parameters (Å^2^)

**Table:**

	*U*^11^	*U*^22^	*U*^33^	*U*^12^	*U*^13^	*U*^23^
Pd1	0.01082 (6)	0.01346 (6)	0.01586 (6)	0.00033 (5)	0.00199 (4)	−0.00143 (5)
Cl1	0.01600 (19)	0.0219 (2)	0.02365 (19)	0.00088 (15)	0.00219 (15)	−0.00764 (16)
P1	0.01157 (19)	0.01407 (19)	0.01712 (19)	−0.00003 (14)	0.00017 (15)	−0.00109 (14)
P2	0.01061 (18)	0.01640 (19)	0.01917 (19)	0.00028 (16)	0.00255 (15)	−0.00093 (16)
C1	0.0188 (8)	0.0192 (8)	0.0188 (8)	0.0020 (6)	0.0049 (6)	−0.0017 (6)
C2	0.0209 (9)	0.0182 (8)	0.0196 (8)	0.0019 (6)	0.0025 (7)	−0.0014 (6)
C3	0.0259 (9)	0.0197 (8)	0.0175 (8)	0.0008 (7)	0.0042 (7)	−0.0034 (6)
C4	0.0362 (11)	0.0236 (9)	0.0209 (8)	0.0085 (8)	0.0056 (8)	−0.0020 (7)
C5	0.0229 (9)	0.0228 (9)	0.0197 (8)	0.0079 (7)	0.0062 (7)	0.0017 (7)
C6	0.0217 (9)	0.0217 (8)	0.0195 (8)	0.0049 (7)	0.0022 (7)	−0.0007 (6)
C7	0.0163 (8)	0.0215 (9)	0.0203 (8)	−0.0024 (6)	0.0011 (6)	−0.0014 (6)
C8	0.0156 (8)	0.0221 (9)	0.0243 (8)	0.0041 (6)	0.0058 (7)	0.0012 (7)
C11	0.0130 (7)	0.0148 (8)	0.0246 (8)	−0.0013 (6)	0.0057 (6)	−0.0009 (6)
C12	0.0247 (9)	0.0215 (9)	0.0319 (10)	0.0015 (7)	0.0097 (8)	0.0040 (7)
C13	0.0167 (9)	0.0281 (10)	0.0312 (9)	−0.0050 (7)	0.0051 (7)	−0.0033 (7)
C14	0.0229 (9)	0.0274 (10)	0.0234 (8)	−0.0038 (7)	0.0074 (7)	−0.0028 (7)
C15	0.0183 (8)	0.0168 (8)	0.0213 (8)	0.0009 (6)	−0.0021 (6)	0.0009 (6)
C16	0.0272 (10)	0.0241 (9)	0.0302 (9)	0.0025 (8)	−0.0097 (8)	0.0021 (8)
C17	0.0263 (10)	0.0181 (9)	0.0315 (10)	0.0055 (7)	−0.0014 (8)	0.0006 (7)
C18	0.0288 (10)	0.0225 (9)	0.0300 (9)	0.0003 (8)	0.0052 (8)	0.0073 (7)
C21	0.0148 (8)	0.0236 (9)	0.0208 (8)	0.0022 (7)	0.0012 (6)	−0.0007 (7)
C22	0.0385 (11)	0.0218 (9)	0.0228 (9)	0.0025 (8)	−0.0020 (8)	0.0023 (7)
C23	0.0180 (9)	0.0516 (13)	0.0254 (9)	0.0069 (9)	0.0000 (7)	0.0019 (9)
C24	0.0240 (9)	0.0258 (9)	0.0209 (8)	−0.0009 (7)	0.0044 (7)	0.0000 (7)
C25	0.0145 (8)	0.0199 (8)	0.0258 (8)	−0.0019 (6)	0.0047 (6)	0.0003 (7)
C26	0.0191 (9)	0.0281 (10)	0.0462 (11)	−0.0043 (7)	0.0120 (8)	0.0042 (9)
C27	0.0234 (9)	0.0204 (9)	0.0363 (10)	−0.0062 (7)	0.0044 (8)	−0.0022 (8)
C28	0.0265 (10)	0.0236 (9)	0.0324 (10)	−0.0019 (7)	0.0046 (8)	0.0078 (8)

### Geometric parameters (Å, º)

**Table:**

Pd1—C1	2.0808 (16)	C14—H14A	0.9600
Pd1—P2	2.3226 (4)	C14—H14B	0.9600
Pd1—P1	2.3233 (4)	C14—H14C	0.9600
Pd1—Cl1	2.4405 (4)	C15—C17	1.526 (2)
P1—C7	1.8352 (17)	C15—C16	1.537 (2)
P1—C11	1.8810 (16)	C15—C18	1.538 (2)
P1—C15	1.8828 (17)	C16—H16A	0.9600
P2—C8	1.8394 (17)	C16—H16B	0.9600
P2—C21	1.8827 (17)	C16—H16C	0.9600
P2—C25	1.8835 (17)	C17—H17A	0.9600
C1—C2	1.530 (2)	C17—H17B	0.9600
C1—C6	1.530 (2)	C17—H17C	0.9600
C1—H1	0.9800	C18—H18A	0.9600
C2—C7	1.522 (2)	C18—H18B	0.9600
C2—C3	1.529 (2)	C18—H18C	0.9600
C2—H2	0.9800	C21—C24	1.532 (2)
C3—C4	1.513 (2)	C21—C23	1.533 (2)
C3—H3A	0.9700	C21—C22	1.546 (2)
C3—H3B	0.9700	C22—H22A	0.9600
C4—C5	1.529 (3)	C22—H22B	0.9600
C4—H4A	0.9700	C22—H22C	0.9600
C4—H4B	0.9700	C23—H23A	0.9600
C5—C6	1.529 (2)	C23—H23B	0.9600
C5—H5A	0.9700	C23—H23C	0.9600
C5—H5B	0.9700	C24—H24A	0.9600
C6—C8	1.522 (2)	C24—H24B	0.9600
C6—H6	0.9800	C24—H24C	0.9600
C7—H7A	0.9700	C25—C27	1.526 (2)
C7—H7B	0.9700	C25—C26	1.534 (2)
C8—H8A	0.9700	C25—C28	1.544 (2)
C8—H8B	0.9700	C26—H26A	0.9600
C11—C13	1.528 (2)	C26—H26B	0.9600
C11—C14	1.533 (2)	C26—H26C	0.9600
C11—C12	1.537 (2)	C27—H27A	0.9600
C12—H12A	0.9600	C27—H27B	0.9600
C12—H12B	0.9600	C27—H27C	0.9600
C12—H12C	0.9600	C28—H28A	0.9600
C13—H13A	0.9600	C28—H28B	0.9600
C13—H13B	0.9600	C28—H28C	0.9600
C13—H13C	0.9600		
			
C1—Pd1—P2	83.84 (5)	C11—C13—H13C	109.5
C1—Pd1—P1	82.82 (5)	H13A—C13—H13C	109.5
P2—Pd1—P1	166.495 (15)	H13B—C13—H13C	109.5
C1—Pd1—Cl1	174.27 (5)	C11—C14—H14A	109.5
P2—Pd1—Cl1	96.201 (14)	C11—C14—H14B	109.5
P1—Pd1—Cl1	96.853 (14)	H14A—C14—H14B	109.5
C7—P1—C11	104.62 (7)	C11—C14—H14C	109.5
C7—P1—C15	104.21 (8)	H14A—C14—H14C	109.5
C11—P1—C15	112.70 (7)	H14B—C14—H14C	109.5
C7—P1—Pd1	103.48 (6)	C17—C15—C16	109.18 (14)
C11—P1—Pd1	116.43 (5)	C17—C15—C18	108.71 (14)
C15—P1—Pd1	113.65 (5)	C16—C15—C18	108.39 (14)
C8—P2—C21	105.91 (8)	C17—C15—P1	110.59 (11)
C8—P2—C25	104.13 (8)	C16—C15—P1	114.69 (12)
C21—P2—C25	111.83 (8)	C18—C15—P1	105.05 (11)
C8—P2—Pd1	102.38 (6)	C15—C16—H16A	109.5
C21—P2—Pd1	117.07 (5)	C15—C16—H16B	109.5
C25—P2—Pd1	113.80 (5)	H16A—C16—H16B	109.5
C2—C1—C6	110.78 (14)	C15—C16—H16C	109.5
C2—C1—Pd1	114.66 (11)	H16A—C16—H16C	109.5
C6—C1—Pd1	114.96 (11)	H16B—C16—H16C	109.5
C2—C1—H1	105.1	C15—C17—H17A	109.5
C6—C1—H1	105.1	C15—C17—H17B	109.5
Pd1—C1—H1	105.1	H17A—C17—H17B	109.5
C7—C2—C3	111.48 (13)	C15—C17—H17C	109.5
C7—C2—C1	110.69 (13)	H17A—C17—H17C	109.5
C3—C2—C1	112.39 (14)	H17B—C17—H17C	109.5
C7—C2—H2	107.3	C15—C18—H18A	109.5
C3—C2—H2	107.3	C15—C18—H18B	109.5
C1—C2—H2	107.3	H18A—C18—H18B	109.5
C4—C3—C2	112.57 (14)	C15—C18—H18C	109.5
C4—C3—H3A	109.1	H18A—C18—H18C	109.5
C2—C3—H3A	109.1	H18B—C18—H18C	109.5
C4—C3—H3B	109.1	C24—C21—C23	109.83 (14)
C2—C3—H3B	109.1	C24—C21—C22	107.91 (14)
H3A—C3—H3B	107.8	C23—C21—C22	108.62 (15)
C3—C4—C5	110.85 (14)	C24—C21—P2	110.58 (11)
C3—C4—H4A	109.5	C23—C21—P2	113.75 (12)
C5—C4—H4A	109.5	C22—C21—P2	105.90 (11)
C3—C4—H4B	109.5	C21—C22—H22A	109.5
C5—C4—H4B	109.5	C21—C22—H22B	109.5
H4A—C4—H4B	108.1	H22A—C22—H22B	109.5
C4—C5—C6	111.11 (14)	C21—C22—H22C	109.5
C4—C5—H5A	109.4	H22A—C22—H22C	109.5
C6—C5—H5A	109.4	H22B—C22—H22C	109.5
C4—C5—H5B	109.4	C21—C23—H23A	109.5
C6—C5—H5B	109.4	C21—C23—H23B	109.5
H5A—C5—H5B	108.0	H23A—C23—H23B	109.5
C8—C6—C5	112.41 (14)	C21—C23—H23C	109.5
C8—C6—C1	110.62 (14)	H23A—C23—H23C	109.5
C5—C6—C1	111.44 (14)	H23B—C23—H23C	109.5
C8—C6—H6	107.4	C21—C24—H24A	109.5
C5—C6—H6	107.4	C21—C24—H24B	109.5
C1—C6—H6	107.4	H24A—C24—H24B	109.5
C2—C7—P1	109.10 (11)	C21—C24—H24C	109.5
C2—C7—H7A	109.9	H24A—C24—H24C	109.5
P1—C7—H7A	109.9	H24B—C24—H24C	109.5
C2—C7—H7B	109.9	C27—C25—C26	109.96 (15)
P1—C7—H7B	109.9	C27—C25—C28	108.08 (14)
H7A—C7—H7B	108.3	C26—C25—C28	108.45 (14)
C6—C8—P2	108.99 (11)	C27—C25—P2	110.88 (12)
C6—C8—H8A	109.9	C26—C25—P2	113.98 (12)
P2—C8—H8A	109.9	C28—C25—P2	105.21 (11)
C6—C8—H8B	109.9	C25—C26—H26A	109.5
P2—C8—H8B	109.9	C25—C26—H26B	109.5
H8A—C8—H8B	108.3	H26A—C26—H26B	109.5
C13—C11—C14	109.63 (14)	C25—C26—H26C	109.5
C13—C11—C12	108.94 (14)	H26A—C26—H26C	109.5
C14—C11—C12	107.99 (14)	H26B—C26—H26C	109.5
C13—C11—P1	113.85 (12)	C25—C27—H27A	109.5
C14—C11—P1	110.77 (11)	C25—C27—H27B	109.5
C12—C11—P1	105.41 (11)	H27A—C27—H27B	109.5
C11—C12—H12A	109.5	C25—C27—H27C	109.5
C11—C12—H12B	109.5	H27A—C27—H27C	109.5
H12A—C12—H12B	109.5	H27B—C27—H27C	109.5
C11—C12—H12C	109.5	C25—C28—H28A	109.5
H12A—C12—H12C	109.5	C25—C28—H28B	109.5
H12B—C12—H12C	109.5	H28A—C28—H28B	109.5
C11—C13—H13A	109.5	C25—C28—H28C	109.5
C11—C13—H13B	109.5	H28A—C28—H28C	109.5
H13A—C13—H13B	109.5	H28B—C28—H28C	109.5
